# What consumers, general practitioners and mental health professionals want: the co-design and prototype testing of a transdiagnostic, acceptance and commitment therapy-based online intervention to reduce distress and promote wellbeing among Australian adults

**DOI:** 10.1186/s12889-023-16688-3

**Published:** 2023-09-14

**Authors:** Donna Hughes-Barton, Gemma Skaczkowski, Chloe Fletcher, Deborah Turnbull, Janne McMahon, Kate M. Gunn

**Affiliations:** 1 IIMPACT in Health, Department of Rural Health, Allied Health and Human Performance, University of South Australia, City East Campus, North Terrace, Adelaide, South Australia 5001 Australia; 2 School of Psychology, The University of Adelaide, Adelaide, SA 5000 Australia; 3 Lived Experience Australia, PO Box 12, Oaklands Park, 5046 Australia

**Keywords:** Mental health, Online intervention, Wellbeing, Acceptance and Commitment Therapy, Website, Web-based, Psychological flexibility, Psychology

## Abstract

**Background:**

Many Australians experience mental health challenges, but only a third access face-to-face psychological services, due to multiple barriers including long waitlists. Additional strategies to prevent or help people de-escalate distress at an early stage are needed. Web-based mental health interventions are becoming increasingly acceptable to consumers and referring General Practitioners (GPs), but most are designed for specific disorders/populations. This study explores consumers’ and health professionals’ preferences and recommendations for the design of a transdiagnostic, Acceptance and Commitment Therapy (ACT)-based, online intervention for Australian adults.

**Methods:**

Thirty-five people (consumers, carers, GPs, mental health professionals) participated in one or more co-design stages. Stage 1: semi-structured interviews to establish what is wanted from such websites (*n* = 22). Stage 2: feedback emailed on branding options (*n* = 20). Stage 3: feedback provided via Zoom or an online survey after testing a website prototype (*n* = 19). Data were analysed using Thematic Framework Analysis and descriptive statistics.

**Results:**

Stage 1 highlighted nine key design principles (plus 25 subthemes) that participants emphasised as important to ensure the website would have broad appeal and meet their needs: (1) user choice is valued highly; (2) ACT-based content is acceptable as it is focused on helping people be proactive and ‘get unstuck’; (3) non-pathologising, direct, empowering, lay language is endorsed; (4) a positive look and feel is appreciated; (5) images and videos are important to break up text and maintain engagement; (6) short text messages to aid engagement are valued; (7) provision of tailored psychoeducation for highly distressed and suicidal users is endorsed; (8) personal and proactive brand name is preferred (*icanactnow*); (9) diverse marketing and training activities are recommended. In Stage 2, *icanactnow* branding preferences were elicited (simplicity, colours to represent growth and a call to action). Stage 3 resulted in the inclusion of a safety plan template and a tailored entry portal for people referred to *icanactnow* by health professionals. High levels of satisfaction with the prototype were reported.

**Conclusions:**

These findings informed *icanactnow* and provide insights for the development of other online mental health interventions, in ways that appeal to both consumers and professionals recommending them.

## Introduction

Mental health problems are commonly experienced by many Australians, and suicide is the leading cause of death in young people under 45 years, and the third leading cause of death in people aged 45–64 years [1]. The economic cost of mental ill-health in Australia is estimated to be $550–600 million per day, including direct health service costs and indirect costs such as lost productivity and reduced life expectancy [2]. Only about a third of people experiencing mental health problems access traditional face-to-face mental health services [3] due to a range of barriers including stigma, unavailability of services, or low levels of mental health literacy [4]. About three quarters of those who do seek help, reach out to their General Practitioner (GP) in the first instance [3]. Reports show that GPs are responding to more mental health-related issues now than in the past [5], as recognition of the value of seeking psychological support is increasing. In a recent report, it was estimated that 38% of all GP consultations addressed mental health issues [6]. However, there is now a great shortage of psychologists and other mental health professionals, and long waiting-lists of 2–6 months are common [2]. Alternate measures are needed that can give people avenues to proactively improve their mental wellbeing, and bridge the wait-time, as well as de-escalate distress and prevent suicide.

Web-based interventions are a possible solution, particularly those that are evidence-based and show efficacy for reducing distress. Internet access is improving its reach across Australia [7, 8] and research shows that many Australians are willing to search for health-related information online [9]. Web-based interventions have high acceptability (e.g., [10–13]), and can be accessed anonymously, any time of the day or night, which may help to overcome both structural or attitudinal barriers to seeking traditional face-to-face support [14].

There is support from Australian GPs for their patients to utilise evidence-based online interventions [15, 16]. Recent studies have demonstrated that web-based interventions can be successfully suggested to patients by health professionals, including GPs, and incorporated into routine mental health and wellbeing care [17, 18]. However, a recent scoping review of 52 web-based resources targeting depression, anxiety, general wellbeing or suicidal ideation that are freely available to Australian adults, revealed that most programs target a specific mental health issue (e.g. depression or anxiety) [19], limiting their reach to distinct diagnostic groups. As detailed in the review [19], *MindSpot* and *This Way Up* are exceptions to this and host programs targeting more than one issue – for example, *MindSpot* offers a range of general wellbeing courses and *This Way Up* offers courses for symptoms of mixed depression and anxiety. In addition, the review found that almost all of the programs identified were based on Cognitive Behavioural Therapy (CBT) [19], which is a problem-focused approach that traditionally focuses on identifying and challenging unhelpful behaviours and thoughts (through a process called cognitive restructuring) [20]. Only one of the interventions included in the review (*ifarmwell.com.au*) was based on Acceptance and Commitment Therapy (ACT); a transdiagnostic, process-oriented approach that targets mechanisms that underlie multiple diagnoses. ACT teaches skills that enable people to accept difficult thoughts and feelings, gain distance (defuse) from them, and find ways to live a values-aligned life, in the present moment, regardless of their circumstances [21]. The processes that underpin ACT (*contact with the present moment*, *acceptance*, *defusion*, *self-as-context*, *values*, and *committed action*) help to build ‘psychological flexibility’ [21, 22], which is considered to be fundamental to emotional health and wellbeing [23, 24]. The evidence base for ACT-based interventions is rapidly expanding; it is considered an efficacious therapeutic approach for the treatment of different mental health conditions, as well as for building resilience and wellbeing in community and workplace settings among the general population [25–29]. In support of this, *ifarmwell*, which was co-developed with farmers, specifically for farmers, has been shown to be highly acceptable to website users [30], and a recent evaluation found that completion of the five modules was associated with a significant reduction in distress and increase in mental wellbeing that was maintained for at least 6 months [31].

Web-based interventions that use a transdiagnostic approach (such as ACT) offer much promise, given that a large proportion of people needing mental health assistance are likely to face multiple diagnoses, and many people dislike diagnoses (or ‘labels’) being attached to their experiences of distress. There is a need for a non-pathologising and transdiagnostic web-based intervention, similar to *ifarmwell*, that is acceptable and applicable to the wider Australian adult population. Due to *ifarmwell’s* impact and acceptability [30, 31], and the growing popularity and evidence base of ACT [25–29], *ifarmwell* was selected as a model to build upon. To aid its translation from an intervention co-designed with farmers to one of relevance to the general adult population, a co-design methodology was adopted to invite the voice of consumers, as well as health professionals who may be likely to refer them to the website. Involving stakeholders in the design of an intervention can enhance trust in the intervention and its acceptability [32], as well as improve the implementation process [33].

For these reasons, key stakeholders, including mental health service consumers, carers, GPs and mental health professionals (psychologists, counsellors, peer workers), were invited to provide their opinion on the design and delivery of an online web-based intervention, to best meet the mental health and wellbeing needs of members of the general adult population. More specifically, it was hoped that this intervention could effectively help to 1) share practical tips with people who want to proactively improve their ability to manage stress and prevent mental health issues; 2) provide evidence-based strategies to people who currently experience poor mental health; and 3) provide guidance to people who are waiting to make an appointment with a mental health professional. This paper describes the outcomes from the co-design process, with a view to aiding the development of future consumer-centred, online mental health interventions.

## Methods

### Study design

**Fig. 1:**
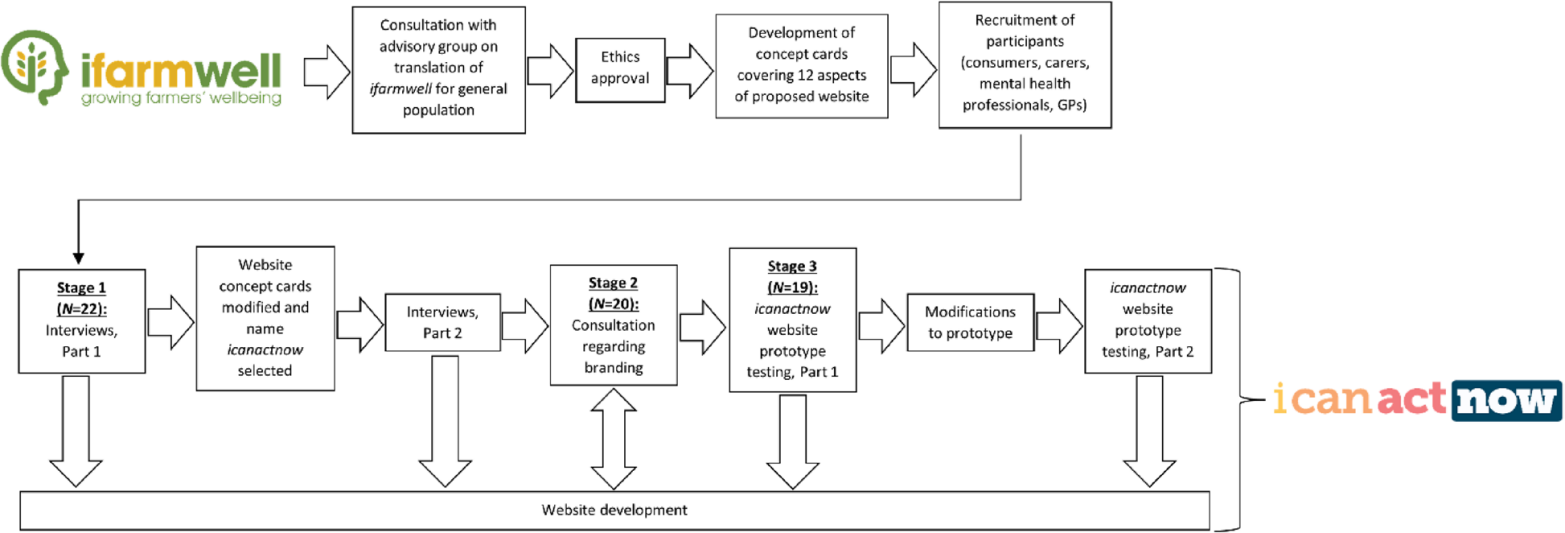
The co-design method

The study was conducted in three stages between September 2020 and December 2022: Stage 1 involved initial co-design interviews where participants reviewed aspects of the proposed website by reflecting on concept cards; Stage 2 involved consulting participants about website branding; and Stage 3 involved participants testing and providing feedback on a prototype of the website. These are depicted in Fig. 1 and described in detail below. Each stage followed an online co-design model that had been adapted to meet changing needs during the COVID-19 pandemic [34]. This was an adaptation of Trischler and colleagues’ co-design framework – an approach that provides guidelines for recruitment and sensitisation of co-design participants, strategies to enable co-design among participants, and advice for implementation of the ideas generated [35].

Methods and results are reported according to the Consolidated Criteria for Reporting Qualitative Research standards for reporting qualitative research [36]. Ethics approval was granted by the University of South Australia’s Human Research Ethics Committee (Project number 203046).

### Recruitment

Three participant groups were involved in the co-design process: (1) an advisory panel of ten experts comprising consumers, GPs and mental health professionals, (2) adult mental health service consumers (including current or potential consumers) and carers, and (3) GPs and mental health professionals (including psychologists, counsellors, and peer workers).

Advisory panel members (*N* = 10) were recruited via personal and professional networks to guide the co-design process. One panel member, KG (Clinical Psychologist), was a Chief Investigator, Chair of the advisory panel, and founder and author of the *ifarmwell* intervention. Further, DT was a Chief Investigator. The remaining panel were selected based on their clinical expertise, knowledge that they could contribute from their own lived experience, and/or leadership in this field. Meetings were held with the panel where they helped inform the scope and focus of the intervention and associated research, and they reviewed the study materials prior to data collection. Nine members of the panel (all except KG) were also invited to participate in each of the three study stages (described in detail below).

Adult mental health service consumers (people who had experienced poor mental health), carers of people who had experienced poor mental health, GPs and mental health professionals (psychologists, counsellors, peer workers) (*N* = 20) were recruited via key community, personal and professional contacts to participate in the co-design process. These participants are referred to hereafter as the ‘original community sample’ and were invited to participate in all stages of the study. The vast majority of consumers and carers who participated were recruited with the help of Lived Experience Australia (a well-established, national mental health consumer and carer advocacy organisation), who put out a call to their membership for study participants. A maximum variation purposive sampling method [37] was employed based upon age and gender to ensure that a variety of demographic characteristics were represented. One participant consented to take part but was later lost to follow up, effectively withdrawing from the study. During Stage 3 of the study, a second sample of community members (consumers, carers, GPs and mental health professionals; *N* = 14) were recruited via a call-out through personal and professional networks and invited to test a prototype of the website (*n* = 6 consented to take part and provided feedback).

**Fig. 2:**
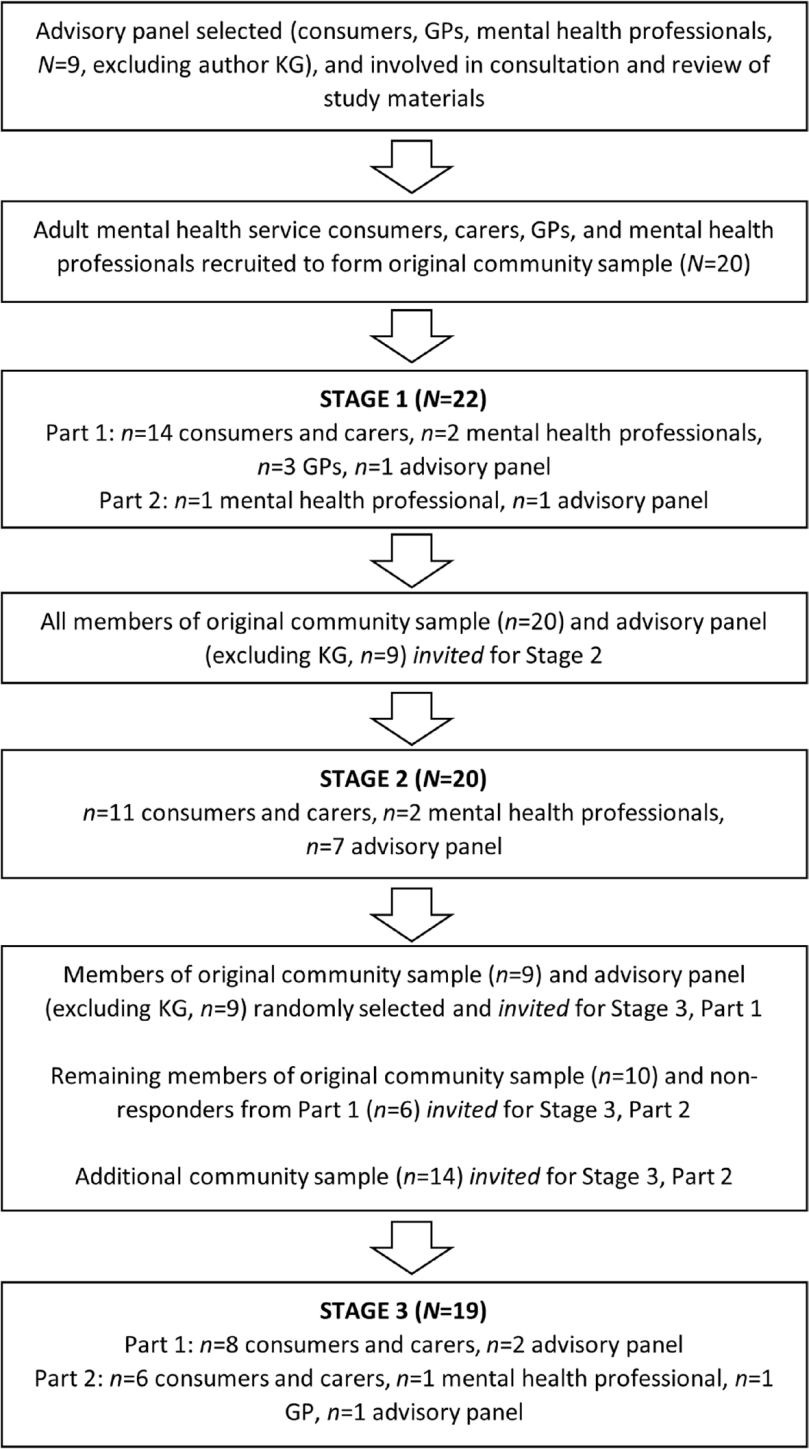
The participants comprising each of the three co-design stages

Thirty-five people participated in one or more stages of the co-design process. This comprised 26 from the community (18 consumers with a range of severity of mental health issues, plus four GPs and four mental health professionals, 58% female), and 9 advisory panel members (three consumers, plus three GPs and three mental health professionals, 78% female). Figure 2 depicts the number of people who were recruited, and participated, in each of the three stages.

### Procedures and materials

Prior to enrolment, potential participants were given an Information Sheet and the opportunity to discuss the study with the researchers. Those who were interested in participating completed an online consent form, and a short questionnaire (online or verbally during the interview) to capture demographic information (age, gender, postcode, marital status, country of birth, highest qualification achieved, current computer and Internet use, and number of hours per week spent on the Internet). In Stage 1, telephone/Zoom interviews were held with each participant, enabling a degree of ‘face-to-face’ contact. Stage 2 was conducted via email, and in Stage 3 participants could choose to provide their feedback via a telephone/Zoom interview or an online survey. Participants who were consumers, carers, GPs, or mental health professionals were offered gift vouchers as partial reimbursement for their time and involvement in each stage of the study. Participants involved in Stages 1 and/or 3 were offered a $100 gift voucher, and participants involved in Stage 2 were offered a $20 gift voucher. Participants involved in more than one stage of the study received multiple gift vouchers as appropriate. Reimbursement was refused by all advisory panel members who participated in the study, although it was offered.

### Stage 1: Co-design interviews

**Fig. 3:**
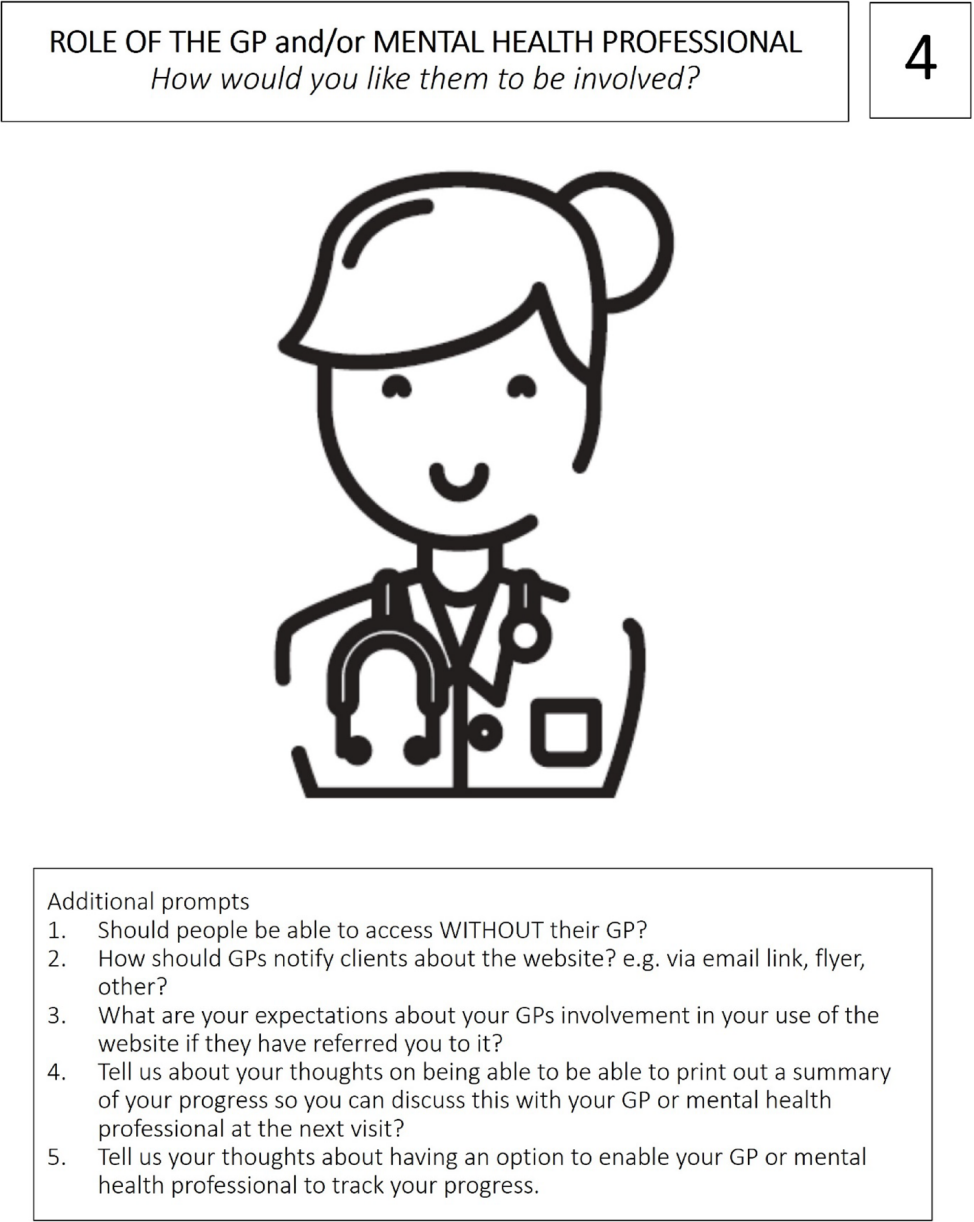
Example of a ‘concept card’ for use to guide Stage 1 interviews

Each enrolled participant was sent 12 pre-reading ‘concept cards’ (sensitisation materials) prior to their semi-structured interview. Each one comprised an A4 page with one topic related to an aspect of the proposed website, a key question for the participant to consider and several sub-questions. The concepts were based on key design features of the *ifarmwell* website that we wanted to ensure were adapted appropriately for a non-farming audience. This pre-sensitisation activity was done to engage participants and initiate thoughts and reflections on the topics, prior to data collection [34, 35]. Table 1 depicts the topics covered on each concept card and each key question, and Fig. 3 provides an example of one of the concept cards.

**Table 1:** Concept card headings, key questions, and changes made for the Part 2 interviews

Concept 1: Tip sheets
***Key question:*** What topics do you think users would like to see covered in Tip Sheets on the website?
***Part 2***^***a***^***:*** Collated topics presented to participants, along with four additional topics for discussion “we are unsure about these potential topics”
**Concept 2: Module content**
***Key question:*** Tell us about your views on the following topics being covered in the modules
**Concept 3: Website structure**
***Key question:*** How would you like to progress through the website / modules?
**Concept 4: Role of the GP and/or mental health professional**
***Key question:*** How would you like them to be involved?
***Part 2***^***a***^***:*** Topics presented for inclusion in a ‘Summary of Progress’ to share with GPs/mental health professionals
**Concept 5: High distress**
***Key question:*** What should we do if someone identifies as highly distressed?
***Part 2***^***a***^***:*** (1) Ideas to date presented with question “These are some ideas to date – any additional suggestions?”; (2) High distress users will also be issued with suicidal ideation screener (PHQ9 [38] within the module: "Have you had any thoughts of actually hurting yourself?" yes/no, followed by P4 screening questions; (3) ‘when and how to seek additional help’ information page to be included; (4) ‘distress button’ to be visible at all times; (5) participants informed that GPs/Allied Health professionals will NOT be notified of users’ high distress or suicidal ideation by the website but users will be encouraged to print off their summary/report and take it to a consult with their GP/Allied Health professional as soon as possible
**Concept 6: Website name**
***Key question:*** Tell us your thoughts on what the website should be named
***Part 2***^***a***^***:*** Website name decided (*icanactnow*) – any other comments on branding?
**Concept 7: Interactivity (text messages)**
***Key question:*** Tell us how you think texts could enhance your experience and engagement with the strategies outlined in the modules
***Part 2***^***a***^***:*** Short, 2–3 times per week, purpose of texts listed, option to write own messages and schedule time for delivery
**Concept 8: Look and feel**
***Key question:*** Tell us how you’d like the website to look and feel
***Parts 2***^***a***^***:*** Friendly and approachable. Inclusion of videos
**Concept 9: Toolbox for consumers**
***Key question:*** Tell us about the type of information you would like to be able to refer back to easily via your personal toolbox/user home page
***Part 2***^***a***^***:*** Ideas collated to date presented to participants for comment
**Concept 10: Language and tone**
***Key question:*** Tell us about the type of language and tone that is appropriate for this website
***Part 2***^***a***^***:*** Non-pathologising, friendly, professional. Avoid ‘mental health’, ‘coping’. Use ‘wellbeing’ (although some consumers dislike this word), ‘managing’ (instead of ‘coping’). Focus on ‘what is important to me’ rather than ‘what is wrong with me’
**Concept 11: Engagement and marketing**
***Key question:*** How should we let people know about the website?
***Part 2***^***a***^***:*** List of ideas collected presented to participants with question “Any other ideas?”
**Concept 12: Metrics**
***Key question:*** Do you want to be able to see website metrics / measures of success?
***Part 2***^***a***^***:*** Participants informed that metrics not clearly endorsed – likely to leave out

^a^Changes made for Part 2 interviews

Stage 1 interviews were conducted in two parts: 14 consumers and carers, two mental health professionals, three GPs, and one advisory panel member (*N* = 20) participated in interviews between September and November 2020 (Part 1), and one mental health professional and one advisory panel member (*N* = 2) participated in interviews in December 2020 and January 2021 (Part 2). Key insights arose from the interviews conducted in Part 1, which informed an updated version of the concept cards that were used in Part 2. For example, the name ‘*icanactnow’* emerged as the consistently preferred name of the website in Part 1, which was tested with participants in Part 2. These changes are depicted in Table 1.

Individual interviews were conducted with each participant at a time that suited them. The interviews were conducted by DHB (either alone or in tandem with GS or KG). All interviewers were female and have a background in psychology and experience in qualitative research methods. Interviews took between 30 and 90 min, were recorded with participants’ consent and were professionally transcribed verbatim. Rapport was established with each participant prior to the interviews. During the interviews, both participant and interviewer(s) referred to the concept cards, and the interviewer used open-ended questions and appropriate probing questions (mainly as listed on the card) when required.

Interviews were continued until data saturation had been reached [39] and no new information arose (*N* = 22). Participants were not provided with transcripts for comment prior to data analysis due to time constraints. However, all participants were invited to take part in the subsequent revision process of the website content (Stages 2 and 3) that had been adapted by CF based on their suggestions. Therefore, they had the opportunity to observe the evolution of the content based on their feedback (and the feedback of other participants) and to ensure that their views and ideas had been translated to the intervention as they intended.

### Analyses of stage 1 co-design interviews

Data were analysed using Thematic Framework Analysis [40, 41] aided by NVivo 12 qualitative data analysis software. Thematic Framework Analysis is a systematic method of categorising and organising qualitative data; a matrix is used to organise data by participant and code, enabling researchers to identify commonalities and differences in the data, and draw descriptive conclusions centred around themes (patterns or concepts that appear across the dataset) [42]. Data analysis followed a pragmatic, descriptive, essentialist approach. This meant that participants’ accounts were viewed as direct insights into their experience [40], rather than being examined as social constructions expressed through language.

Transcripts were anonymised and uploaded into NVivo. Each was read and re-read. Data corresponding to consumers’ preferences for a mental health and wellbeing-related website were highlighted. An inductive or ‘bottom-up’ approach was employed to ensure that participants’ experiences were adequately captured, and although the original concept cards guided much of the discussion, broad themes unrelated to specific concept cards were also generated. Meaning was situated at the semantic or surface level of the data. Themes were reviewed and discussed between DHB and KG until consensus was reached [40].

### Stage 2: Consultation regarding website branding

**Fig. 4:**
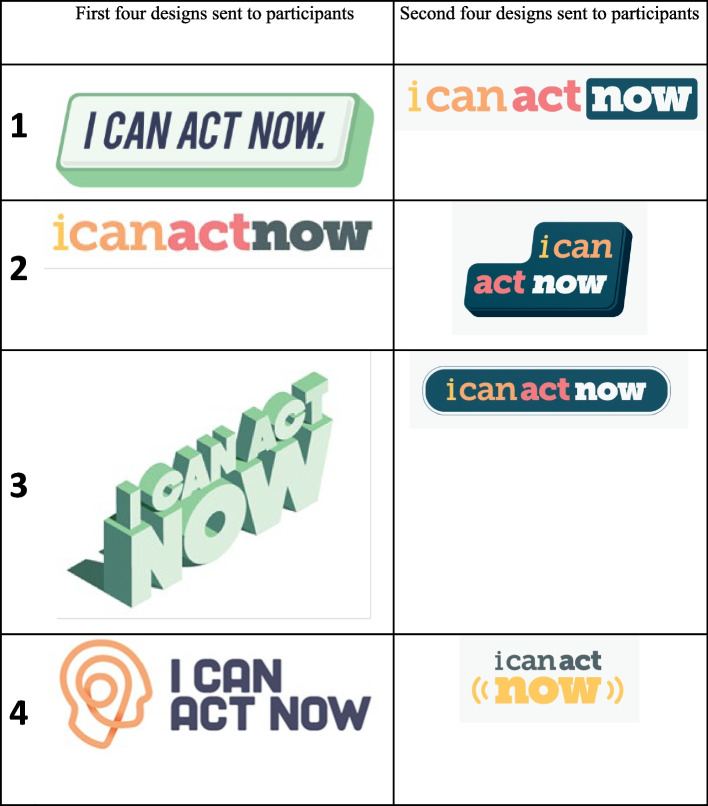
Co-design Stage 2 branding consultation: Four initial potential logo designs

In March 2021, all members of the original community sample and the advisory panel were invited to provide their opinion on branding options for the website. Four potential designs were sent to each participant via email, with feedback provided via return email. The first column of Fig. 4 shows the four logo options provided to participants. Feedback was received from 11 consumers and carers, two mental health professionals, and seven advisory panel members, and was then provided to the graphic designers, who incorporated it into an additional four logos that were reviewed again by the same participants. These are shown in the second column of Fig. 4.

### Stage 3: Prototype testing

Prior to public launch of the website, a prototype was made available by the web developers. Content was informed by Stage 1 participant feedback and incorporated the Mindframe guidelines for image and language use [43, 44]. The Mindframe guidelines inform use of imagery and language that aims to minimise stigma and harm (e.g., through use of non-judgemental and non-stigmatising language), maximise diversity of representation (e.g., through use of a diverse range of images), break down misinformation (e.g., through use of language that informs rather than alarms), and promote help-seeking (e.g., through use of language that is empowering) [43, 44].

All members of the original community sample and the advisory panel were emailed and invited to register and spend about 60–90 min working through and testing aspects of the website between June and November 2022. Prototype testing was conducted in two parts, with modifications made to the website in response to feedback from participants in Part 1 and prior to testing by participants in Part 2. Members of the original community sample and the advisory panel were randomly selected and invited to participate in either Part 1 or Part 2 of the prototype testing. In Part 1, nine members of the original community sample and nine members of the advisory panel were invited to test the prototype website. Key changes were made to the prototype website based on participants’ feedback. In Part 2, the remaining 10 members of the original community sample were invited to take part, and six participants who had not responded to the initial invitation (in Part 1) were sent a second invitation to participate. A further 14 community members who had no previous knowledge of the website were also invited to test the prototype, to provide additional, independent insights. Again, key changes were made to the prototype website by CF (in consultation with KG where necessary) based on participants’ feedback. Feedback on the prototype website was provided by 14 consumers and carers (including eight from the original community sample and six from a second sample of community members), one mental health professional, one GP, and three advisory panel members.

Feedback was provided via online survey or interview (at each participant’s discretion), with data collected using the same methods in both Parts 1 and 2. Each participant was provided with the survey questions prior to website testing to give them a framework to guide their feedback. The survey comprised a qualitative and quantitative component. The qualitative survey component included 14 questions. Participants were invited to provide their opinion on seven aspects of the website that corresponded to the key design features highlighted on the concept cards that guided the Stage 1 interviews (i.e., overall impression, registration process, visuals, language, interactive components, navigation through the website, portals for consumers to share their progress with a friend or for health professionals to share the site with clients). For each of these seven aspects, participants were prompted to indicate what they liked about it, and then to suggest anything that could be improved. In the quantitative component of the survey, participants completed the System Usability Scale [45], which is a 10-item validated measure used to assess usability of the website. Each of the 10 items (e.g., *I think I would like to use the icanactnow website*) was rated by participants on a 5-point Likert measure ranging from Strongly disagree (0) to Strongly agree (4). Participants were also asked about their likelihood of referring others to *icanactnow* in the future by indicating their response on a sliding scale from Not at all likely (0) to Extremely likely (100). They were also asked to complete five demographic questions (occupation/work role, gender, age group, postcode, and familiarity with other mental health and wellbeing websites). Participants who chose to have an interview completed the qualitative components via interview and quantitative components via online survey.

## Results

### Stage 1: Co-design interviews

Twenty-two people participated in the Stage 1 interviews (*n* = 14 consumers and carers, *n* = 3 mental health professionals, *n* = 3 GPs, *n* = 2 advisory panel; 59% female). They were aged between 26 and 83 years (*M* = 50.9, *SD* = 14.5). Over half (12/22; 55%) had a post-graduate degree, were living with a partner (13/22; 59%) and most were born in Australia (19/22; 86%). All used the internet each week for a range of activities including email, social media, banking, shopping, weather, work, research and entertainment (range 12–42 h, *M* = 25.9, *SD* = 10.2).

Twelve concept topics were covered in the interviews and from these, nine consistent key design principles, or themes (with 25 subthemes), were generated. These are summarised below and illustrated by one or two key quotes. Subthemes and additional quotes are provided in Table 2. Themes included:

1. User choice is valued highly

Findings generally indicated that consumers wanted some degree of flexibility, choice, tailoring and the opportunity for personalisation in module content. It was suggested that these attributes could help users remain engaged with the intervention. For example, they were supportive of being in control of how frequently they could engage with content and the flexibility to save progress to continue with later. Tailoring and choice could also be connected to the contents of a personalised toolbox of learned skills, tips and tools that users could refer back to in the future. Mixed opinions arose around the addition of jokes and inspiring quotes, but the ability to turn these on and off could give users control over their inclusion. Flexibility and personalisation could also be aligned with the type of information to share with others, including support for personalised, printable summaries or discussion prompts to take to appointments with health professionals (or show to family and friends), the opportunity for a health professional or friend to support their progress, and the opportunity to be sent a text message with the contact details of key support people in times of distress.

*“I think with whatever tool you're going to make, it's important that the person can make it their own.” (ID11, Consumer).*

 

(2) ACT-based content is acceptable as it focused on helping people be proactive and get unstuck

Participants were presented with the proposed titles of the five modules of the intervention, which were based on ACT and were adapted from *ifarmwell*. These five titles were: Taking stock of your current wellbeing and some practical strategies to get you started (Module 1), Thoughts are like bullies – how to spend less time ‘in your head’ (Module 2), Getting the most out of life/working out what is important (Module 3), Shifting your attention to the present (Module 4), Putting it all together and moving forward (Module 5). Participants recommended non-pathologising, direct, empowering and lay language, and supported the modules’ focus and components as tools to help people reduce distress and move forward in their lives. The focus of the modules’ content on ‘what’s important to me’ (not ‘what’s wrong with me’) was supported by each participant as non-pathologising and a proactive way to help them connect with their values. In addition, the intervention contains exercises to help raise users’ awareness of their unhelpful thoughts and ‘stories’ that may colour their perception. Consumers and health professionals saw value in being able to identify these as a means to move forward. Health professionals added that they were also in favour of consumers bringing a summary document to appointments because it demonstrated patient proactiveness and could save consultation time. Participants also saw value in a website that enabled the user to monitor their own symptoms and progress.

*“Also, identifying some of the thoughts and stories, too. Because that can be hard for people. That having the, this is what’s important to me and this is what’s getting in the way.” (ID08, Consumer)*

The next three themes were concerned with the look and feel of the website content. These are listed, with an illustrative quote, and then summarised.

(3) Non-pathologising, direct, empowering, lay language is endorsed

*“You want the everyday person to use this. Always remember to speak in their language.” (ID17, Consumer)*

(4) A positive look and feel is appreciated

*“So it needs to be formal in that it's well-structured and clear and broken up into sections and people don't have to mess around to find what they have to find and it all works well, but it needs to sort of look casual and not like a really regimented website from something that you would have had to do at school or something.” (ID18, Consumer)*

(5) Images and videos are important to break up text and maintain engagement

*“… my ability to read when I'm unwell and to be able to retain information… you need to sort of tailor for both because there'll be people that can read and then other people that just find that really difficult to read and retain stuff.” (ID13, Consumer)*

Participants reported that a mix of text and imagery, lived-experience videos, and positive, non-pathologising language as well as a ‘professionally informal’ appearance would appeal to most future users of the site. There were mixed opinions about colour choices although all described their chosen colours as positive; some participants preferred bright colours that were perceived as stimulating, and others preferred softer colours perceived as calming. Participants were also mixed in their opinion of using, and personalising, avatars; some thought this facilitated personalisation of the intervention, whereas others thought that this might draw attention away from the intervention itself.

(6) Short text messages to aid engagement are valued

A key feature of the proposed website was that consumers provide their mobile number at registration and remain engaged with the website and its materials via automated text messages sent to them at varying times throughout the intervention. Participants agreed that text messages could function as a ‘check-in’, to keep users engaged and remind them that they are involved in the intervention. They were supportive of frequent encouraging text messages and reminders of goals and to practice new skills, including the ability to take ownership of one’s own progress by writing themselves texts and reminders.

*“… reminding you of key concepts and reminding you to keep going, reminding you to use strategies after you’ve done it.” (ID07, Consumer)*

(7) Provision of tailored psychoeducation for highly distressed and suicidal users is endorsed

Participants raised concerns about how highly distressed users would be managed. The GPs in the cohort were generally not in favour of being contacted if a client was highly distressed, due to concerns over availability and where responsibility lay, especially if a contact message was missed. In addition, it was raised that not all GPs are comfortable or experienced with dealing with distress, and most are time-poor. This could all lead to a variable, non-reliable source of support. However, participants endorsed the provision of psychoeducation about the nature of suicidal thoughts to help de-escalate distress. Providing users with tailored information about key specialised, free acute 24-h mental health services and helplines was valued, as was encouraging users who identify as highly distressed to make an appointment with a GP or mental health professional. Participants recommended that these services include those that are text-based in case a user cannot speak due to distress. It was also suggested that this information could be sent via text message to the user, and it was felt that the act of receiving such a text could be therapeutic in itself. A further recommendation was for contact details of users’ own personal key contacts to be texted to them so that phone numbers could be accessed easily and dialled directly in cases where speech and dexterity were affected by extreme distress.

*“… the difficulty with that is what happens to that information? What if I don’t – for example, what if I don’t, (a) I don’t receive the information, (b) I’m not around to be able to act on it, where is that left? You know what does it mean then?” (ID21, GP)*

*“ ...have available those numbers for the normal suicide, Lifeline hotline, Kids Helpline that sort of stuff.” (ID10, Mental Health professional).*

(8) Personal and proactive brand name is preferred (icanactnow).

Participants were presented with a list of 12 potential names for the website. They reported a preference for names that included the user (i.e., ‘I’ or ‘my’ or ‘me’), to empower and personalise the user’s journey with the intervention. They suggested that a name containing ‘act now’ was proactive and encouraging of them taking action to improve their mental health, especially while waiting to see a mental health specialist.

*“I just think it's a bit more active...These are the things that I can do when I feel distressed.” (ID15, Consumer)*

(9) Diverse marketing training activities are recommended.

Participants provided ideas for marketing avenues when the new website is ready for roll-out, agreeing that multiple concurrent methods may be most efficient.

*“I think Google optimisation, social media, referrals from GPs, and I think getting in the media would be a good idea. So whether it would be on radio stations or podcasts or things like that would be really good, and community groups as well” (ID09, Consumer) *

**Table 2:** Themes and subthemes identified from interviews of Stage 1, using Thematic Framework Analysis

**Themes** Subthemes	**Example quote(s)**
**Theme 1: User choice is valued highly**	
Must be personalised/ feel as though it is tailored specifically for them	*“I'd probably go for the more personal approach. Because after all, it is about you. If you were having a conversation with a psychologist, that's how your name would be used, wouldn't it?”* (ID02, Consumer)
Want to be able to choose what information they share about themselves and what aspects of the content they engage with	*“I think you asked whether people would provide either/or, so either email or phone number, or if they want to provide—they can tick both and they can provide both. That becomes their option.”* (ID12, Consumer) *“I think some people find it really spooky that the computer always seems to know you and know what you’re doing and what you’re thinking, you know? So, I think both, having the option of being able to call yourself by name or being able to do something else maybe would be nice.”* (ID14, Mental Health professional)
Want to be able to choose how frequently they engage with content	*“Flexibility, I think, is important, because everybody is different and you're never going to get it right all of the time.”* (ID02, Consumer) *“… if the material is flexible enough that because maybe some people want to get through it one module a day, and maybe some people want to do one module a week or a month. But let people—give people the option how, of what they want—of when they want to set them.”* (ID16, Consumer) *“Yeah look I guess it depends on the individual doesn't it. Is there an option to ask a person how frequent they would like them?”* (ID13, Consumer) *“… if you’re unwell, the mind can feel overloaded very quickly and you might want to do things in very small portions.”* (ID14, Mental Health professional)
Want to be able to choose the information they share with their health professionals and others about their involvement	*“… I don’t know if they can remove bits or decide depending on which GP they see what they can share*.” (ID014, Mental Health professional) *“… because some people still like some privacy in some areas. They still don't like to tell everything.”* (ID06, Consumer) *“Psychologists are great, that'll go that with you, but I'd love it if people could use family and friends more. That's what we need in life. The system's just clogged up with so many people.”* (ID11, Consumer)
Want to be able to choose whether or not jokes are presented	*“Jokes, I love it. Give more and more. I'll have them, thank you very much*.” (ID02, Consumer) *“Look it's hard isn't it because if you've got an individual who's really struggling, a joke can just sort of go – ‘you know what, they think this is funny’. It's not funny. Yeah.”* (ID13, Consumer) *“Including jokes, I think jokes should definitely be optional. Maybe opt-out.”* (ID16, Consumer) *“… it’s very hard to find jokes that don't offend people, and I think little one or two liner dad jokes are probably good things to lighten the load throughout.”* (ID10, Mental Health professional)
Support for the inclusion of a personal toolbox	*“Yeah, I love the toolbox ideas.”* (ID11, Consumer) *“…yeah, it’s a great idea to be able to refer back to the toolbox.”* (ID16, Consumer)
Consumers want flexibility with text message frequency	*“Some people might just want to have something to checks in with them once a day, then they can decide from there.”* (ID04, Consumer) *“I mean, if it's—it's different for someone who says they're distressed and then you send them those messages and then check in with them to ask them how they're going. That sort of thing [unclear] for that person, so it's a little bit subjective, but, yeah, I mean, it's probably good to follow that same system as ifarmwell, if that works for them.”* (ID18, Consumer) *“Yeah, I think reminders are a good idea. But in think opt-in reminders are a better idea than the blanket ones.”* (ID16, Consumer)
Choice and personalisation will aid engagement	*“So that’s what I mean by the flexibility, because it said how to engage people, and I think so they don’t leave early, because if it's too confronting, they might leave early. If it's too confusing, if it's patronising, I think they are the things that tend to encourage people to disengage or for the reasons people disengage.”* (ID12, Consumer)
**Theme 2: ACT-based content is acceptable as it is focused on helping people be proactive and ‘get unstuck’**	
Appreciate focus on ‘what’s important to me’ not ‘what’s wrong with me’	*“Knowing what floats your boat, what drives your mood, and what your values are. Why, because it reminds yourself to get back on track with the things that you love.”* (ID17, Consumer) *“’What’s the matter with me’ is a very negative kind of connotation, isn’t it? Yeah. Oh god. I’ve got something wrong with me. Well, I can be even more depressed now.”* (ID02, Consumer)
Value in becoming aware of their thoughts and ‘stories’, their impact and learning how to ‘unhook’ from them	*“I really like this – the thoughts in your head because I think that’s where a lot of people get stuck whether they’re anxious, depressed, stressed, whatever, the stuff that’s going around in your head and the thoughts that you have I think are the hardest to deal with and to know how to get them out. I thought that was particularly helpful and fairly upfront as well.”* (ID07, Consumer) *“I liked the way that it was more focused on wellbeing and particularly around thinking and the power that negative thoughts have.”* (ID01, GP)
Generation of a summary of intervention progress to share with health professionals is a good idea, can prompt discussion	*“Yeah I think that’s brilliant because it’s a really good tool isn’t then to form a discussion and it’s optional.”* (ID13, Consumer) *“I think that’s a great idea … when I go to my psychologist or my GP, I’m just trying to pull back memories of what I’ve been doing and all the rest of it, rather than if you’ve got a printout, you’ve got some stuff you’ve been going through, some specific things to talk through.”* (ID09, Consumer) *“I’d be more than happy for a patient to bring me something. I’d be pleased actually, because it shows that they’re doing something.”* (ID21, GP) *“… you can show your important person in your life, like your mum, your partner, you could also show it to your GP as well.”* (ID11, Consumer)
Favour inclusion of self-assessments to help them monitor symptoms and progress	*“… from my own experience, with having mental health for so long, I’m very conscious about day-to-day and what my triggers are and things like that. So I think having those things will help people identify them and help their quality of life.”* (ID09, Consumer)
Toolbox highlight community services and support that may assist with recovery, not just clinical services	*“… list of places that are normally in everybody’s community like you know library, at places where you can be social… If you’re feeling lonely even just walking in a shopping centre and being around people can help.”* (ID12, Consumer) *“… not just medical or clinical, but things that people can do with their time in their community that do make them feel good like community gardens and activities… To encourage socialising.”* (ID15, Consumer)
**Theme 3: Non-pathologising, direct, empowering, lay language is endorsed**	*“… certainly not academic language, because we could lose people in that.”* (ID02, Consumer) *“…we should talk about our mental health in the same way that we talk about our physical health. I speak to my patients a lot about the brain being an organ like any organ and we can become unhealthy anywhere, including with our mental health and we shouldn’t be afraid to talk about it….”* (ID21, Mental Health professional)
**Theme 4: A positive look and feel is appreciated**	
Bright colours are stimulating and positive	*“Because they're just something for the eyes to be stimulated with, stimulate your mind.”* (ID04, Consumer) *“I suppose when people are going through this type of stuff they’re probably going to be either in a mellow state or a depressive state, or something like that. So I would probably think some brighter colours through it, just to shed some light.”* (ID09, Consumer)
Softer colours are calming	*“I’d probably be tending to go towards the calmer colours… like the mauves, blues, greens rather than reds and yellows.”* (ID14, Mental Health professional)
Mixed opinion on inclusion of avatars	*“I think the avatars are good, I think it adds a bit of fun to it, and letting people design their own avatars also gives them some personalisation and ownership of it, so I welcome that.”* (ID09, Consumer) *“I've been saying, have wacky avatars, ones with armour plating on them. As you're getting more resilient, I'm getting building up my armour, I'm getting tougher. Or an angel that gets a bigger halo and stuff.”* (ID11, Consumer) *“I get you're trying to be friendly and approachable and stuff, but I think people want direct and they want to act now. They don't want bullshit of I didn't come to pick a character. I just think some people might respond like that.”* (ID18, Consumer) *“… you could get bogged down in that or not make it past that section.”* (ID07, Consumer)
A ‘professionally informal’ look will appeal to most	*“If it's too formal, then you might not catch everybody. You're going to get a lot of variety of people, so you want it not to be too formal, otherwise you won't catch the people that, maybe they do struggle reading, and things like that. Too many big words might put them off.”* (ID06, Consumer) *“… you have to make it approachable, and you have to make it as comfortable for them as possible, but obviously it still needs to be really well structured and put together and achieve what it needs to achieve.”* (ID18, Consumer)
**Theme 5: Images and videos are important to break up text and maintain engagement**	*“… maybe breaking it down a bit more and using infographics or a little quick video or something might break it up a bit more but still get all the information across.”* (ID09, Consumer) *“ … I think if you were feeling overwhelmed or English was your second language or you had intellectual difficulties, that you probably wouldn't even bother reading all that.”* (ID15, Consumer)
Preferences for lived experience videos	*“Yeah, see he looks like me. Just a regular dude. You don't want clinicians on there, you don't want people in ties. Every time you go to a conference, the people that really hit you hard are the lived experience talkers.”* (ID11, Consumer) *“I think it's different when you're showing vignettes of people talking about their experiences… I think people engage with that. They know it's a real person.”* (ID19, Mental Health professional)
**Theme 6: Short text messages to aid engagement are valued**	
Useful check-ins	*“I think that can be good sometimes … Like,’Hi, we haven't seen you for a while. Just wondering how you're travelling? We really hope you're doing well or’—but always positive, because we don't need negativity.”* (ID02, Consumer) *“… it lets people know you're still interested in them.”* (ID10, Mental Health professional)
Useful reminders	*“Yeah, and you know maybe even is there a reason, you know, has something gotten in the way of your progress that you need to seek some—talk to someone. Talk to a real person.”* (ID13, Consumer)
Welcome idea of writing their own text messages to themselves, to enable them to take ownership and remind themselves of their goals and plans	*“I think that's a good idea to encourage them to put it into their diaries as like a recurring reminder because that way, they are becoming—I don't know what's the word for it? Where they take ownership of it and do it themselves rather than rely on the app to do it because in a couple of weeks, they'll be finished with this program, but you want them to continue these habits ongoing.”* (ID15, Consumer)
**Theme 7: Provision of tailored psychoeducation for highly distressed and suicidal users is endorsed**	
Need to advise users of sources of support in addition to this web-based resource (possibly via text in addition to displaying the information on the screen)	*“ … if patients have the information around all of the distress and suicide helplines and ED, like the ACIS – you know the triage line, mental health triage – that really is the service for suicidality, really, and high levels of distress. So I think it’s about making sure it’s directed appropriately”* (ID21, GP) *“ … what have you got to lose trying it. I think it—I reckon it could actually do good. In that moment, that split second, they go someone cares, even if it's a computer. It's like someone cares about me. I guess that's all one really needs when they're going through those—someone's listening.”* (ID17, Consumer) *“But people always turn to websites in distress. I think the worst thing about [a current website] is you do this series of questions and then the next minute you're, contact a mental health professional and it's like, well it's midnight, I can't do that. So, I think they need to be—the numbers absolutely need to be 24 h type help.”* (ID13, Consumer) *“ … you offer the phone numbers of their support person or their online services; whether there's a text-based one or the phone ones because they might be in a public place and they might not want to talk on the phone.”* (ID15, Consumer)
Remind users of supportive people within their own personal network	*“Part of setting up the thing is to actually nominate one or two people that can be a buddy. Because obviously in mental health people jump to the medical, people need acute whatever, but no, not everyone in distress does need that. Sometimes they just need connection.”* (ID01, GP) *“When you're stressed, you can't even find those numbers… Yeah, but also, you can't even like hit a keyboard when you're very stressed. People's dexterity is affected, so I think that's a good idea.”* (ID15, Consumer)
Provision of psychoeducation on the management of suicidal thoughts	*“In my view, education is everything. Allow people to be aware that their suicidal thoughts are just thoughts, and it's not them. It's not who they are, although it's coming from their own mind, but they don't have—for me, education is everything. That's how I got to where I am today.”* (ID17, Consumer) *“I'd prefer us to frame suicidal ideation as just unhelpful coping … the thought of that provides some comfort or relief or escape or something like that. So if you're going to provide—I suppose you would provide some sort of ACT-consistent psychoeducation about it, directing them back into the resources you're already providing, then probably that would be—I'd be supportive of that.”* (ID19, Mental Health professional)
GPs uncomfortable with being notified if their patient has scored as highly distressed or suicidal	*“…once the GP knows, the GPs then got a moral responsibility, because they’ve got an established relationship with the patient, and that could end up causing a huge amount of angst and distress for the GPs I think.”* (ID01, GP) *“These people need more professional help. There is no point in saying the GPs are going to provide it. Because we're not psychologists.”* (ID23, GP) *“I think it comes back to the particular GP. So if we were to pick a GP who felt less confident in managing mental health related stuff – for example some of the – and again this isn’t about discriminating against people who have been trained in other countries, but I think it’s important to bear in mind that there are cultural differences and different levels of confidence in dealing with stuff like that … I think it would probably need to be followed up with the offer of some training for GPs in how they might then manage that situation.”* (ID20, GP) *“… part of my problem is that I’ll have limited time. I don’t provide focus psychological services. So it’s more just the 15 to 30 min service.”* (ID21, GP)
**Theme 8: Personal and proactive brand name is preferred (** ***icanactnow*** **)**	*“I quite like the “I” because I feel like it gives people that little bit of power back. People often feel vulnerable and unempowered when they’re mentally unwell.”* (ID21, GP) *“I think Act Now looks best and is the most serious and is appealing because people, if they’re accessing that service, they want to act now and they want to get some help now, because the problem is that you can’t get into a psychologist straightaway, so I think that makes sense from that perspective.”* (ID18, Consumer)
**Theme 9: Diverse marketing and training activities are recommended**	*“… the National Mental Health Consumer and Carer Forum. Lived Experience Australia. Mental Health Australia put out a newsletter. Who else? Carers SA. There's a national carers organisation as well. I can't remember what they're called. Then there's the Consumers Health Forum, the National Consumers Health Forum.”* (ID13, Consumer) *“It probably would need to be kind of multiple points of attack. Some advertising in medical media, so through oh there’s Medical Observer and Australian Doctor, RSCGP, ACRRM all of those, they’ve got websites.”* (ID01, GP)

### Stage 2: Consultation regarding website branding

Twenty participants took part in the website branding consultation (*n* = 11 consumers and carers, *n* = 2 mental health professionals, *n* = 7 advisory panel; refer to Fig. 2). The first four logos (see Fig. 4) were emailed to each participant for comment. Three themes arose from their responses, summarised below with illustrative quotes.

1. An appreciation for simplicity

Although participants reported different logos as their first choice, a common theme arose highlighting their preference for a logo that is simple and easy to read.

*“Number 2 is my preference because it’s simple, sleek, modern, and it makes the individual words stand out.” (ID07, Consumer)*

*“I prefer No. 1 – it is simple and uncomplicated. My second choice is No. 4, again because it’s simple.” (ID10, Mental health professional)*

(2) A liking for colours that represent growth

Participants also commented on the colour choices of the sample logos. The colours of Logo 2 were particularly liked because they blended well and represented personal growth as a user works through the intervention.

*“FAVE - Modern, colourful, I like progression from light colours to darker (confidence growing) to 'act' in black, bold font (strong).” (ID15, Consumer)*

*“I like this one more. The different colours are quite impactful..… assertive?” (ID22, Mental health professional)*

(3) A preference for a logo to represent ‘I can take action now’

Participants’ comments also indicated that a logo that represented movement or a ‘call to action’ was important to them.

*“I like [number] 1 because it looks like a button that people can push for help, though I'd remove the full stop and play with changing the colours. My vote is 1.” (ID18, Consumer)*

*“I also like number 4. Creative and smart. I don’t think it is the correct font, it feels as though it has no “call to action”…and is more a pondering thought. It doesn’t tell me to do what it is asking.” (ID17, Consumer)*

*“[Number 3 is] good… seems on the move.” (ID22, Mental health professional)*

Following this feedback, four more sample logos were created (see Fig. 4) and shown to the participants who responded to the first branding consultation round. Feedback was received by 13 (13/19; 68%) and indicated a preference for number 1.

*“I prefer logo no.1 it blends well and is more modern and professional looking.” (ID06, Consumer)*

The final logo was then decided upon and created, as seen in Fig. 1.

### Stage 3: Website prototype testing

As shown on Fig. 2, 19 people participated in the website prototype testing phase (*n* = 14 consumers and carers, *n* = 1 mental health professional, *n* = 1 GP, *n* = 3 advisory panel). Prototype testing was conducted in two parts. Part 1 participants reported spending between 30 min and ‘one day’ testing aspects of the website. As a result of the feedback from participants in Part 1, the following changes were made to the prototype: buttons were enlarged and a banner was added to the homepage to clarify purpose of the website and encourage registration; buttons were added to ‘I need a hand’ and ‘What to do in a crisis’ pages to break up text and assist navigation; additional illustrations were included to break up text and encourage engagement; a safety planning template was developed and included as part of tip sheets offered to users; an information page was added for users referred to *icanactnow* by their GPs; greater diversity (e.g., gender, age, cultural background) was added to imagery and interactive elements throughout the website (e.g., imagery on homepage, illustrations in modules, and avatar options); and a message for young people aged under 18 years that included links to age-appropriate mental health support websites was added to the ‘Register’ page. In addition, the idea of inclusion of lived experience videos was strongly endorsed among the participants. Minor changes were also made to written content throughout the website in response to participant feedback. These included: explanation about the purpose of questionnaires completed as part of the modules; and supportive messages with links to distress services appearing at key trigger points throughout the modules.

Part 2 participants reported that they spent between one and six hours testing the website. Only a few changes were suggested by participants in this phase. These included: further refinement to imagery and illustrations to improve representation of diversity (e.g., disability); changes to banner text on website homepage to refer to different aspects of the modules (e.g., letting go of unhelpful thoughts, doing what matters most, focusing on the present moment); and greater clarity around the registration process (e.g., need for mobile phone number).

All participants were asked to indicate on a scale of Not at all likely (0) to Extremely likely (100) “How likely are you to recommend *icanactnow* to someone?” Seven participants responded to the question in Part 1 and eight in Part 2 (15/19, 79%), with results showing high likelihood that *icanactnow* would be recommended to others. Results ranged from 23 to 100, with a median of 100 (Part 1 range 23–100, *M* = 86.1; median 100; Part 2 range 51–100, *M* = 84.6; median 88.5). Sixteen participants (16/19; 84%) completed items on the System Usability Scale [45]. Across both Part 1 and Part 2 of data collection, 15 of these 16 participants indicated high levels of satisfaction with the website by indicating Strongly agree (4) to items such as *“I think I would like to use the icanactnow website”, “I thought the icanactnow website was easy to use”,* “*I would imagine that most people would learn to use the icanactnow website very quickly”*, and *“I felt very confident using the icanactnow website*”; and Strongly disagree (0) to items such as *“I found the icanactnow website unnecessarily complex”* and *“I found the icanactnow website very cumbersome (awkward) to use”.*

The website will be prepared ready for launch in 2023.

## Discussion

This paper describes the development of *icanactnow.* This is a website, co-designed for the general Australian adult population, that was adapted from an existing, effective, theory and consumer-driven website, co-designed with Australian farmers (*ifarmwell*) [31]. The findings from this study support the perceived utility and acceptance of a resource, developed with consumers, and informed by evidence-based strategies, to help adults reduce distress, proactively improve their ability to manage stress and suicidal thoughts, and get more out of life. In addition, the findings supported the development of a web-based resource that provides guidance to people waiting to obtain an appointment with a mental health professional.

*icanactnow* is unique because most currently available web-based resources focus on a particular mental health issue rather than taking a transdiagnostic approach [19]. In addition, the proposed website is unique with its foundation in ACT rather than CBT, which is the basis of most currently available web-based interventions [19]. A main difference between these two therapeutic approaches is that traditional CBT teaches users to challenge unhelpful thoughts, while ACT teaches users to build ‘psychological flexibility’, by helping them accept what they cannot control, gain distance (defuse) from unhelpful thoughts, and focus their attention on values-consistent behaviours, to make the best of their situation [21]. This approach is thought to be particularly useful when the challenges that someone is facing are beyond their control. For example, recent research has shown that psychological flexibility may have helped to protect adults against suicide risk during the COVID-19 pandemic [46].

Involving key stakeholders in the co-design process helps to ensure that the intervention will better fit consumers’ needs [47], be more attractive to those referring them (e.g., GPs and mental health professionals), and is therefore more likely to be used. The development of *ifarmwell* was done in this way [48] and the resultant website has high acceptability among its target audience (farmers) [30].

The co-design process for *icanactnow* demonstrated that consumers, GPs and mental health professionals alike were supportive and enthusiastic about the development of such a website for the general population, provided that key features were incorporated into its design and flow. These key features included a non-pathologising focus on ‘what’s important to me’; providing distressed users with key 24-h phone and text-based options to help them reach out when in need; positive, bright and empowering language and colour; and incorporating a mix of text, imagery and video (preferably featuring people who had lived experience of distress). Another important feature included allowing users’ choice where possible, in personalising their journey through the intervention. Personalisation could be incorporated for example, into the name the user chooses for themselves, the creation of a personalised avatar, inclusion of jokes and inspiring quotes, choice of a printable summary to share with a health professional or personal contact, and the ability to write and schedule their own text messages to remind or motivate themselves. Features of choice and personalisation also arose as important during the co-design interviews with farmers for the development of *ifarmwell* [48]. This focus on choice by both farming and general cohorts is unsurprising, as there is a growing body of evidence demonstrating that personalising or tailoring materials is a key driver of effectiveness within web-based interventions [49].

Both *icanactnow* and *ifarmwell* co-design participants recommended aspects of flexibility and choice, but sometimes the specifics differed. For example, both groups wanted the inclusion of positive and relevant imagery. For the *ifarmwell* codesign participants, this meant green colours and positive farming imagery, leaning towards masculine pictures [48]. In comparison, the *icanactnow* cohort did not unanimously suggest specific colours, and some preferred bright colours while others preferred soft colours. These comparisons highlight that while commonalities are emerging for what people want in an online intervention (e.g., personalisation, choice, non-pathologising look and feel), different audiences have unique requirements, and it may be difficult to find a ‘one size fits all’ approach. Employing a careful co-design process as we have done in the present study, will help bring these unique requirements to the fore.

Another key personalisation feature recommended by the participants for *icanactnow* was the inclusion of their own personal key contacts as sources of support when distressed. Participants suggested that users could enter the names and phone numbers of their own supports at registration. If they identify as distressed while working through the intervention, they could be sent these names and numbers to prompt them to call. These practical suggestions complement the large amount of empirical evidence highlighting the important role that social support plays in preventing and treating poor mental health [50], and endorsing the inclusion of social support in interventions [51].

The co-design process also provided insights into potential dissemination methods. The participants suggested a multi-pronged approach to dissemination that included promotion through social media, community networks and through medical media and networks. In this way, information about the website could be accessed by many potential users as well as by referrers. Internet ‘push’ mechanisms (e.g., digital triggers, reaching out to potential users, rather than waiting for them to search online) may be effective in marketing to potential users through social media or Google optimisation [52]. Future research should test the reach of these strategies, intervention uptake among the general population, as well as the intervention’s impact on users’ distress levels.

This study was not without limitations. As with all research, the consumers, carers, mental health professionals, and GPs who elected to participate likely had a pre-existing interest in mental health and developing novel interventions to improve mental health and wellbeing. Despite this, their comments and suggestions have facilitated the development of an intervention that could have greater reach to all health professionals, including GPs, regardless of their experience or interest in mental health, and to consumers and carers who would not otherwise seek support. The applicability of these findings to the design of websites in settings beyond Australia, or for children and adolescents under 18 years of age, is also unknown, and beyond the scope of the current work.

The strengths of the study lie in its rigorous methodology and the potential application of the development process to other online mental health interventions. A further strength is that the consumer population was selected via a purposive sampling method based upon gender and age. Moreover, the intervention is based on a transdiagnostic approach (ACT) that can be helpful both for people who currently experience poor mental health and those who want to proactively learn skills to improve their general wellbeing and ability to manage stress in their daily lives. The findings of this study are timely. Due to the recent COVID-19 pandemic, and increasing awareness of the value of seeking help, there has never been a more pressing need to provide free mental health support to the general adult population and those waiting to access psychological services.

## Conclusion

Much can be learnt from consumers and carers, GPs and mental health professionals to inform the development of new mental health resources and interventions. This paper not only highlights important consumer and carer-informed design principles for other intervention developers, but also provides an example of how co-design principles can be practically applied to the development of web-based intervention. A range of design principles that are valued by consumers and carers were identified in this process, including suggestions for language and layout, tip and tool inclusions and colours. However, contextual factors that may lead to differences in needs and preferences between population groups should be carefully considered when applying these findings.

Further work is needed to evaluate the impact of the *icanactnow* intervention and to identify effective strategies to disseminate such interventions at scale. Given widespread mental health challenges, unmet needs, mental health workforce shortages and increasing digital literacy in Australia, successful dissemination of online mental health tools such as *icanactnow* has the potential for broad social and economic benefits.

## Data Availability

The authors declare that data supporting the findings of this study are available within the article.

## Abbreviations

ACT: Acceptance and Commitment Therapy
GP: General Practitioner
